# Hypertension care cascade at a large urban HIV clinic in Uganda: a mixed methods study using the Capability, Opportunity, Motivation for Behavior change (COM-B) model

**DOI:** 10.1186/s43058-021-00223-9

**Published:** 2021-10-20

**Authors:** Martin Muddu, Isaac Ssinabulya, Simon P. Kigozi, Rebecca Ssennyonjo, Florence Ayebare, Rodgers Katwesigye, Mary Mbuliro, Isaac Kimera, Chris T. Longenecker, Moses R. Kamya, Jeremy I. Schwartz, Anne R. Katahoire, Fred C. Semitala

**Affiliations:** 1 Makerere University Joint AIDS Program (MJAP), Kampala, Uganda; 2 Uganda Initiative for Integrated Management of Non-Communicable Diseases (UINCD), Kampala, Uganda; 3 Department of Internal Medicine, Makerere University College of Health Sciences, P.O. Box 7072, Kampala, Uganda; 4 Uganda Heart Institute, Mulago Hospital Complex, Kampala, Uganda; 5 Infectious Disease Research Collaboration (IDRC), Kampala, Uganda; 6 Child Health and Development Centre, Makerere University College of Health Sciences, Kampala, Uganda; 7 Case Western Reserve University School of Medicine, Cleveland, OH USA; 8 Section of General Internal Medicine, Yale School of Medicine, 333 Cedar Street, New Haven, CT 06511 USA

**Keywords:** Hypertension and HIV integration, Barriers, Facilitators, Uganda, COM-B

## Abstract

**Background:**

Persons living with HIV (PLHIV) receiving antiretroviral therapy (ART) have a high prevalence of hypertension (HTN) and increased risk of mortality from cardiovascular diseases. HTN and HIV care integration is recommended in Uganda, though its implementation has lagged. In this study, we sought to analyze the HTN and HIV care cascades and explore barriers and facilitators of HTN/HIV integration within a large HIV clinic in urban Uganda.

**Methods:**

We conducted an explanatory sequential mixed methods study at Mulago ISS clinic in Kampala, Uganda. We determined proportions of patients in HTN and HIV care cascade steps of screened, diagnosed, initiated on treatment, retained, and controlled. Guided by the Capability, Opportunity, Motivation and Behavior (COM-B) model, we then conducted semi-structured interviews and focus group discussions with healthcare providers (*n* = 13) and hypertensive PLHIV (*n* = 32). We coded the qualitative data deductively and analyzed the data thematically categorizing them as themes that influenced HTN care positively or negatively. These denoted barriers and facilitators, respectively.

**Results:**

Of 15,953 adult PLHIV, 99.1% were initiated on ART, 89.5% were retained in care, and 98.0% achieved control (viral suppression) at 1 year. All 15,953 (100%) participants were screened for HTN, of whom 24.3% had HTN. HTN treatment initiation, 1-year retention, and control were low at 1.0%, 15.4%, and 5.0%, respectively. Barriers and facilitators of HTN/HIV integration appeared in all three COM-B domains. Barriers included low patient knowledge of HTN complications, less priority by patients for HTN treatment compared to ART, sub-optimal provider knowledge of HTN treatment, lack of HTN treatment protocols, inadequate supply of anti-hypertensive medicines, and lack of HTN care performance targets. Facilitators included patients’ and providers’ interest in HTN/HIV integration, patients’ interest in PLHIV peer support, providers’ knowledge and skills for HTN screening, optimal ART adherence counseling, and availability of automated BP machines.

**Conclusion:**

The prevalence of HTN among PLHIV is high, but the HTN care cascade is sub-optimal in this successful HIV clinic. To close these gaps, models of integrated HTN/HIV care are urgently needed. These findings provide a basis for designing contextually appropriate interventions for HTN/HIV integration in Uganda and other low- and middle-income countries.

Contributions to the literature

- We utilized the widely used and validated COM-B model to assess determinants of integrated HTN/HIV care in the urban setting
- To our knowledge, this is the first mixed methods study to analyze the hypertension care cascade in an urban HIV treatment program and explore barriers and facilitators to HTN/HIV integrated care using the COM-B model.
- The barriers and facilitators will explain why HTN-HIV integration has stalled and inform contextualized implementation strategies to improve it in resource-limited settings.

## Introduction

Persons living with HIV (PLHIV) on antiretroviral therapy (ART) have a high prevalence of hypertension (HTN) compared with the general population [1–4]. In Uganda, approximately 1/3 of PLHIV aged ≥18 years have HTN, a leading cause of cardiovascular disease (CVD) [1, 4–11] and end-stage renal disease in PLHIV [12].

To preserve the gains made in HIV care, the treatment cascade must now be extended to include integration of care for HTN within existing HIV services for dual HTN and HIV control [13–17]. This was tested in a multi-center trial in rural Uganda which demonstrated that an integrated HTN/HIV care model that leverages the HIV infrastructure is preferable to vertical programs for both conditions [16].

Although integration of HTN/HIV care is recommended by the World Health Organization (WHO) and adopted by the Uganda Ministry of Health (MoH) HIV treatment guidelines [18, 19], implementation of these guidelines is sub-optimal in routine settings [20–22].

Despite significant gains towards the UNAIDS 90-90-90 goals of identifying PLHIV, access to effective ART and HIV control [23], the quality of care for HTN among PLHIV is still low in SSA [24, 25]. For instance, in rural Uganda, only 27% of adult PLHIV ever had their blood pressure measured in 2017, and among those treated for HTN, only 24% achieved HTN control [24, 26].

In this rural setting, key barriers to integrated HTN/HIV care included poor access to HTN medications, low provider knowledge of HTN care, inappropriate task shifting, lack of evidence-based treatment protocols, and weak systems for monitoring and evaluation [21, 26–29].

However, the practice of integrated HTN and HIV care and related barriers and facilitators in the urban populations of PLHIV remain less understood.

In this study, we sought to analyze the care cascades for HTN and HIV among adult PLHIV at a large urban HIV clinic in Uganda in order to identify care gaps. In addition, we explored the barriers and facilitators of integrating HTN and HIV care through qualitative inquiry guided by the Capability, Opportunity, Motivation and Behavior (COM-B) model [30, 31]. Understanding the implementation barriers and facilitators would reveal why integrating HTN and HIV has been unsuccessful, and inform the development of well-contextualized strategies to improve it.

## Methods

### Study design

This was an explanatory sequential mixed methods study that was designed based on the “Taxonomy of Mixed Methods Design in Implementation Research” by Palinkas et al. [32]. We sequentially collected and analyzed quantitative and qualitative data, beginning with quantitative data. We used quantitative data to determine the performance/outcomes of integrated HTN-HIV care cascades. Qualitative data then contextualized the quantitative findings by elucidating the barriers and facilitators of implementing integrated HTN-HIV care (Table 1). For the quantitative component of the study, we retrospectively collected data to map out the HTN care cascade among adult PLHIV at the Mulago immune-suppressive syndrome (Mulago ISS) clinic in order to identify care gaps.

**Table 1:** Taxonomy of mixed methods design for the study (adopted from Palinkas et al.)

Element	Category	Definition
**Mixed method structure**	Sequential QUAN͢qual	Sequential collection and analysis of quantitative and qualitative data, beginning with quantitative data, for the primary purpose of determining the integrated HTN-HIV care cascades.
**Mixed methods function**	Expansion	We used quantitative data to determine the performance/outcomes of integrated HTN-HIV care cascades and qualitative data to elucidate the barriers and facilitators of implementing integrated HTN-HIV care.
**Mixed methods process**	Connecting the data	The qualitative data set built upon the quantitative data set. The quantitative data set determined the integrated HTN-HIV care cascades and identified the successes and performance gaps. The qualitative data set contextualized the quantitative data by exploring the perceived barriers and facilitators of integrated HTN-HIV care.

For the qualitative, we conducted focus group discussions (FGDs) and semi-structured in-depth interviews (IDIs) with purposively selected hypertensive PLHIV to explore their understanding, beliefs, and perceptions regarding HTN/HIV integrated care. In addition, we conducted semi-structured key informant interviews with healthcare providers at the Mulago ISS clinic to understand their perspectives and experiences regarding integrated HTN/HIV care.

**Fig. 1:**
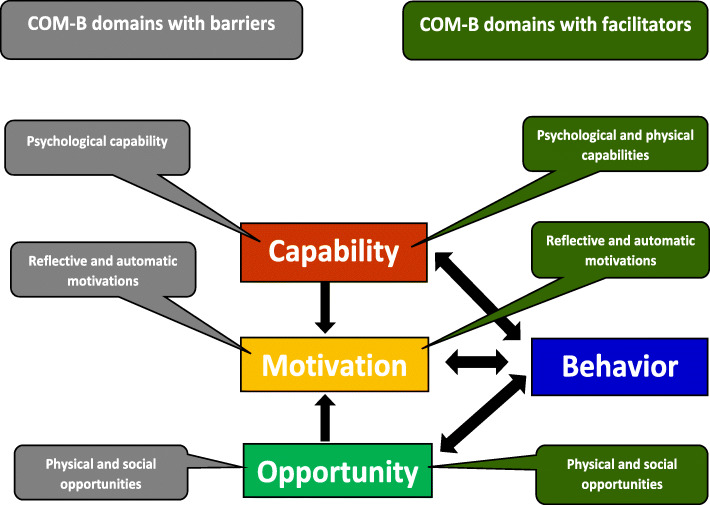
The Capability, Opportunity, Motivation influences Behavior (COM-B) domains which revealed either barriers or facilitators of integrating HTN management into HIV care

We utilized the Capability, Opportunity, Motivation - Behavior (COM-B) model to explore barriers to and facilitators of HTN/HIV integration [30, 31]. We chose the COM-B model, which forms part of the Behavior Change Wheel framework, to understand the capabilities, opportunities, and motivations for patients and providers with regard to integration of HTN management into HIV care [30]. The central principle for the COM-B is that changing any behavior requires changing capability, opportunity, and/or motivation to perform that behavior [31]. Thus, the COM-B model provided a coherent basis for exploring barriers to and facilitators of integrating HTN and HIV care [30] (Fig. 1).

We followed the consolidated criteria for reporting qualitative research (COREQ) guideline in developing this manuscript [33].

### Study setting

We conducted the study at Mulago ISS, the largest HIV clinic in Uganda providing comprehensive HIV services to over 16,500 PLHIV. The clinic is located within Mulago National Referral and Teaching Hospital Complex in Kampala, Uganda’s Capital City. HIV-related clinical activities included HIV testing and counseling (HTC) and management of antiretroviral therapy (ART). The clinic also implemented differentiated ART delivery models where stable PLHIV (good adherence to ART with sustained HIV viral suppression) received their ART refills in the community. Mulago ISS clinic is owned and operated by the Makerere University Joint AIDS Program (MJAP).

At this clinic, HIV services are provided by doctors, nurses, clinical officers, HIV counselors, laboratory technicians, pharmacy technicians, and record officers as well as PLHIV expert clients.

In line with the national guidelines for HIV care, PLHIV were routinely screened for non-communicable diseases including HTN. If a PLHIV was diagnosed with HTN, the clinician typically prescribed both ART and anti-hypertensive medicines at the same time and gave the client one future appointment for both conditions. Prior to the commencement of this study, the clinic had already achieved universal screening for HTN among all PLHIV during each clinic visit. All available medicines and services at the clinic were provided at no cost to the patients. However, patients were advised to pay for medicines, which were out of stock in the clinic, at private pharmacies of their preference. This study was one of the Learning, Implementation, Networking, Knowledge and Support (LINKS) programs funded by Resolve to Save Lives, a US-based non-governmental organization that supported countries globally to improve HTN control. The grant recipient and project implementer in Uganda was MJAP. This formative study was conducted to inform the design of an implementation strategy to integrate HTN and HIV care at this clinic.

### Quantitative study

#### Study participants and sampling

We included PLHIV who were (1) ≥18 years old; (2) enrolled into HIV care at the clinic before May 1, 2018; and (3) had at least one clinic visit between July 1, 2019, and January 1, 2020. We chose this group to enable us to review follow-up data for at least 1 year after ART initiation.

#### Data collection

We extracted retrospective data from the electronic medical records (EMR) for all PLHIV who met the inclusion criteria. The EMR at Mulago ISS clinic uses the Uganda MoH open medical records system which captures patient data on HIV care. The clinic EMR was adapted to include additional data elements on HTN and other non-HIV comorbidities. SPK, MMB, MM, and IK designed an Excel-based instrument which MB and IK used to extract demographic information and clinical data for both HIV and HTN. The data collection tool was developed to obtain information on the HTN care cascade at the Mulago ISS clinic and specifically map out the expected outcomes at each cascade step according to national HIV guidelines and WHO recommendations. The team double-entered the data for quality assurance. After cleaning the data, it was exported to Stata (version 13) for analysis. Throughout the quantitative and qualitative components of this study, hypertension was defined as “having a documented blood pressure (BP) ≥ 140/90 mmHg at least twice one month apart or documented use of HTN medications or documented history of hypertension” [29]. Data on indicators at every stage of the HTN and HIV care cascades were recorded. The steps of screening, diagnosis, treatment initiation, retention, monitoring, and control for HTN and HIV care cascades at the clinic were quantified as adapted from our previous work in Eastern Uganda [29]. Each cascade step was described as a proportion of the prior step and reported as follows for HIV and HTN: number and percentage of patients screened, diagnosed, treated, retained, monitored, and controlled.

#### Data analysis

We conducted univariate analyses to describe socio-demographic and other characteristics of the cohort. Means and standard deviations (*SD*s) were used to describe continuous variables, and percentages and frequencies were used for categorical variables. We then stratified the data into two sub-populations: participants that had HIV alone and those that had both HIV and HTN. We compared baseline characteristics of the two subgroups using *X*^2^ or Fisher exact tests for categorical characteristics and *t* tests for continuous characteristics.

We conducted descriptive analyses of the frequencies and percentages of patients at each previously defined step compared with the preceding step. We then stratified the cascades by HIV (participants with HIV alone) and HIV and HTN (participants with both HIV and HTN). We analyzed the data using Stata (version 13).

### Qualitative study

#### Study participants and sampling

We purposively selected PLHIV with HTN as a sub-set of participants in the quantitative study and healthcare providers caring for PLHIV who had been in the clinic for at least 1 year for the interviews. PLHIV with HTN at Mulago ISS clinic were eligible if they had been on ART for at least 1 year and did not have a cognitive disorder that precluded their active participation in an interview. We approached patients through telephone calls close to their next scheduled clinic appointment.

Eligible healthcare providers were individuals of different cadres who had been providing care to patients at the Mulago ISS clinic for at least 2 years. We approached healthcare providers face to face. We recruited participants until we achieved data saturation. The research assistants completed a contact summary form to indicate any new information they received from each interview and focus group discussion. Data saturation was achieved when no more new information emerged from the interviews and focus group discussions.

#### Data collection

We used semi-structured interview guides and focus group discussion guides based on the three COM-B domains of Capability, Opportunity, and Motivation [30, 31]. All interviews had open-ended questions reflecting patients’ and healthcare providers’ perceptions and perspectives regarding HTN/HIV integration. Prior to data collection, we pretested the interview guides with hypertensive PLHIV and healthcare providers at the Mulago ISS clinic who were not participating in the study. Pretesting helped us to simplify the language in some interview questions which seemed unclear to participants. However, we did not change the meaning. FA, RS, and IA shared the objectives of the study with clinic leaders, healthcare providers, and patients who were contacted to participate and conducted the FGDs, semi-structured IDIs, and KIIs. FA, IA, and RS are all trained social scientists with expertise in qualitative research including conducting FGDs and semi-structured interviews. The interviewers established a relationship with the clinic leaders, healthcare providers, and patients prior to study commencement, but were not part of the healthcare team at the clinic, thus limiting potential bias. We conducted four FGDs with each group consisting of six to eight hypertensive PLHIV. Each session lasted 60 min. FGDs explored PLHIV’s experiences of accessing HTN care and related challenges, alternative care seeking, and recommendations for service improvement. We conducted six semi-structured IDIs with hypertensive PLHIV each lasting 30 min. The patient semi-structured IDIs explored individual lived experiences and perceptions regarding HTN care in the HIV clinic. In addition, we conducted 13 semi-structured KIIs with healthcare providers at the clinic. The semi-structured KIIs explored healthcare providers’ experience with providing HTN care in the HIV clinic, gaps in HTN care, training needs for staff, and recommendations for improvement. Throughout the interviews and FGDs, the researchers employed empathy and were non-judgmental and non-directive. All KIIs were conducted in English while IDIs and FGDs were conducted in Luganda the local language. All interviews were audio-recorded and transcribed verbatim in Luganda and then translated into English.

#### Data analysis

A research team with expertise in public health, social science, and clinical care (FA, MM, and RS) conducted the thematic content analysis. The team coded transcripts using a deductive approach guided by the COM-B as a coding framework. The coding process was guided by consensual qualitative research (CQR) procedures [34]. First, each team member read three transcripts independently and identified preliminary codes. Through a series of meetings, the team agreed on an initial set of codes. To organize and manage the large set of data, all transcripts were coded in Atlas.ti (version 8) software. FA checked all transcripts for accuracy and completeness before they were uploaded into Atlas.ti (version 8) software. IK independently coded eight of the transcripts which FA had coded to establish inter-coder reliability (kappa 0.80). Through more meetings, researchers resolved the discrepancies and developed the final codebook. The codes were then categorized into sub-themes, and these were mapped onto the COM-B domains and constructs.

Code reports were generated centrally; FA synthesized the findings and summarized them. Themes that negatively influenced HTN treatment in the HIV clinic denoted barriers, and those that positively influenced HTN treatment denoted facilitators (Table 4).

We extracted specific quotations from the transcripts to illustrate verbatim expressions of matters that appeared important.

This study was approved by The AIDS Support Organization (TASO) Institutional Review Board (IRB) and the Uganda National Council for Science and Technology (UNCST). Written informed consent was obtained from each participant of the qualitative study.

#### Data validation and feedback to study participants

We conducted a continuing medical education session/workshop with all healthcare providers in the clinic, shared preliminary results of this study, and received feedback. In addition, we shared study findings with patients during health education talks when they came to the clinic for regular follow-up visits. The patients and healthcare providers felt that the results were representative of their experiences and perceptions of integrated HTN/HIV care. There was high demand for integrated HTN/HIV care among participants.

## Results

### Quantitative study

#### Characteristics of study participants

Between July 2019 and January 2020, 15,953 PLHIV were enrolled in the cohort (68% female and mean (*SD*) age of 41 (9.7) years).

Overall, the mean baseline CD4 cell count was 365.3 (*SD* = 292.3) cells per mm^3^. Nearly half of all participants, 7258 (45.9%), were taking tenofovir/lamivudine/efavirenz (TDF/3TC/EFV) as the initial antiretroviral therapy (ART) regimen. A total of 7088 (47%) were overweight or obese (body mass index >25; Table 2).

**Table 2:** Baseline characteristics of the study participants

Characteristic	Overall cohort (*n* = 15,953)	HIV (*n* = 12,061)	HIV and HTN (*n* = 3892)	***P*** value
Age, yrs	40.7 (*SD* = 9.7)	39.4 (*SD* = 9.2)	44.7 (*SD* = 10.4)	<0.001
Age categories, yrs				<0.001
18 to 30	1983 (12.4%)	1728 (14.3%)	255 (6.6%)	
31 to 50	11,458 (71.8%)	8892 (73.7%)	2566 (65.9%)	
Over 50	2411 (15.1%)	1349 (11.2%)	1062 (27.3%)	
Sex				<0.001
Male	5077 (31.8%)	3521 (29.2%)	1556 (40.0%)	
Female	10,876 (68.2%)	8540 (70.8%)	2336 (60.0%)	
Baseline BP, mm Hg				
Systolic	119.2 (*SD* = 47.5)	115.5 (*SD* = 49.3)	145.7 (*SD* = 15.9)	<0.001
Diastolic	77.0 (*SD* = 12.8)	74.7 (*SD* = 11.4)	93.8 (*SD* = 9.9)	<0.001
Baseline ART regimen				<0.001
TDF-3TC-EFV	7258 (45.9%)	5918 (49.7%)	1340 (34.5%)	
AZT-3TC-NVP	4227 (26.7%)	2929 (24.6%)	1298 (33.4%)	
AZT-3TC-EFV	1801 (11.4%)	1306 (11.0%)	495 (12.7%)	
TDF-3TC-NVP	1054 (6.7%)	752 (6.3%)	302 (7.8%)	
Others	1470 (9.3%)	1015 (8.5%)	455 (11.7%)	
Duration on ART				<0.001
< 2 years	3638 (23.0%)	2885 (24.2%)	753 (19.4%)	
2 to 5yrs	5512 (34.9%)	4453 (37.4%)	1059 (27.2%)	
5 to 10yrs	5675 (35.9%)	4004 (33.6%)	1671 (43.0%)	
> 10yrs	985 (6.2%)	578 (4.9%)	407 (10.5%)	
Baseline CD4 count	365.3 (*SD* = 292.3)	365.9 (*SD* = 292.1)	363.6 (*SD* = 293.2)	0.674
Baseline CD4 count by category				0.162
<50	2110 (13.2%)	1610 (13.4%)	500 (12.9%)	
50–<100	1041 (6.5%)	760 (6.3%)	281 (7.2%)	
100–<200	2123 (13.3%)	1591 (13.2%)	532 (13.7%)	
>200	10,657 (66.9%)	8080 (67.1%)	2577 (66.3%)	
Baseline BMI,				0.130
Underweight (<19.0)	1221 (8.1%)	887 (7.8%)	334 (9.0%)	
Normal weight (19.0 to <25.0)	6774 (44.9%)	5122 (45.0%)	1652 (44.6%)	
Overweight (25.0 to <30.0)	4175 (27.7)	3159 (27.8%)	1016 (27.4%)	
Obese (≥30.0)	2913 (19.3)	2210 (19.4%)	703 (19.0%)	

#### Hypertension comorbidity

The prevalence of HTN among PLHIV over 18 years old was 24.3% (30.7% among males and 21.5% among females). Mean baseline systolic and diastolic BP among hypertensive PLHIV were 145.7 (*SD* = 15.9) mmHg and 93.8 (*SD* = 9.9) mmHg, respectively. Females were less likely to have hypertension compared to males [*OR* = 0.65; *95% CI* 0.60–0.71]. PLHIV aged 31 to 50 and those above 50 years were more likely to have HTN than younger patients [*OR* = 3.01; *95% CI* 1.51–6.01], [*OR* = 7.08; *95% CI* 3.53–14.19]. Similarly, HTN was more likely among PLHIV on ART for more than 10 years compared to those on ART for less than 2 years [*OR* = 1.56; *95% CI* 1.30–1.87]. PLHIV whose initial ART regimen contained nevirapine (NVP) were 50–60% more likely to have HTN compared to those whose initial ART contained EFV (Table 3).

**Table 3:** Association between hypertension and sex, age, ART regiment, and duration on ART among PLHIV at the Mulago ISS clinic

Risk factor	Univariate		Multi-variate	
	Odds ratio (***95% CI***)	***P*** value	Odds ratio (***95% CI***)	***P*** value
**Sex**				
Male	*1*	*Ref*	*1*	*Ref*
Female	0.64 (0.59–0.68)	<0.001	0.65 (0.60–0.71)	<0.001
**Age category**				
18–30 years	*1*	*Ref*	*1*	*Ref*
31–50 years	7.32 (3.76–14.26)	<0.001	3.01 (1.51–6.01)	0.002
Over 50 years	19.76 (10.11–38.64)	<0.001	7.08 (3.53–14.19)	<0.001
**Baseline ART regimen**				
TDF-3TC-EFV	*1*	*Ref*	*1*	*Ref*
AZT-3TC-EFV	1.65 (1.47–1.86)	<0.001	1.04 (0.91–1.20)	0.557
TDF-3TC-NVP	1.74 (1.51–2.01)	<0.001	1.54 (1.31–1.81)	<0.001
AZT-3TC-NVP	1.92 (1.76–2.09)	<0.001	1.64 (1.47–1.83)	<0.001
Other	1.78 (1.57–2.01)	<0.001	1.31 (1.13–1.52)	<0.001
**Duration of ART**				
<2 years	*1*	*Ref*	*1*	*Ref*
2 to 5 years	0.91 (0.82–1.01)	0.081	0.78 (0.70–0.87)	<0.001
5 to 10 years	1.60 (1.45–1.76)	<0.001	0.99 (0.88–1.12)	0.893
>10 years	2.70 (2.32–3.13)	<0.001	1.56 (1.30–1.87)	<0.001

#### HIV care cascade

**Fig. 2:**
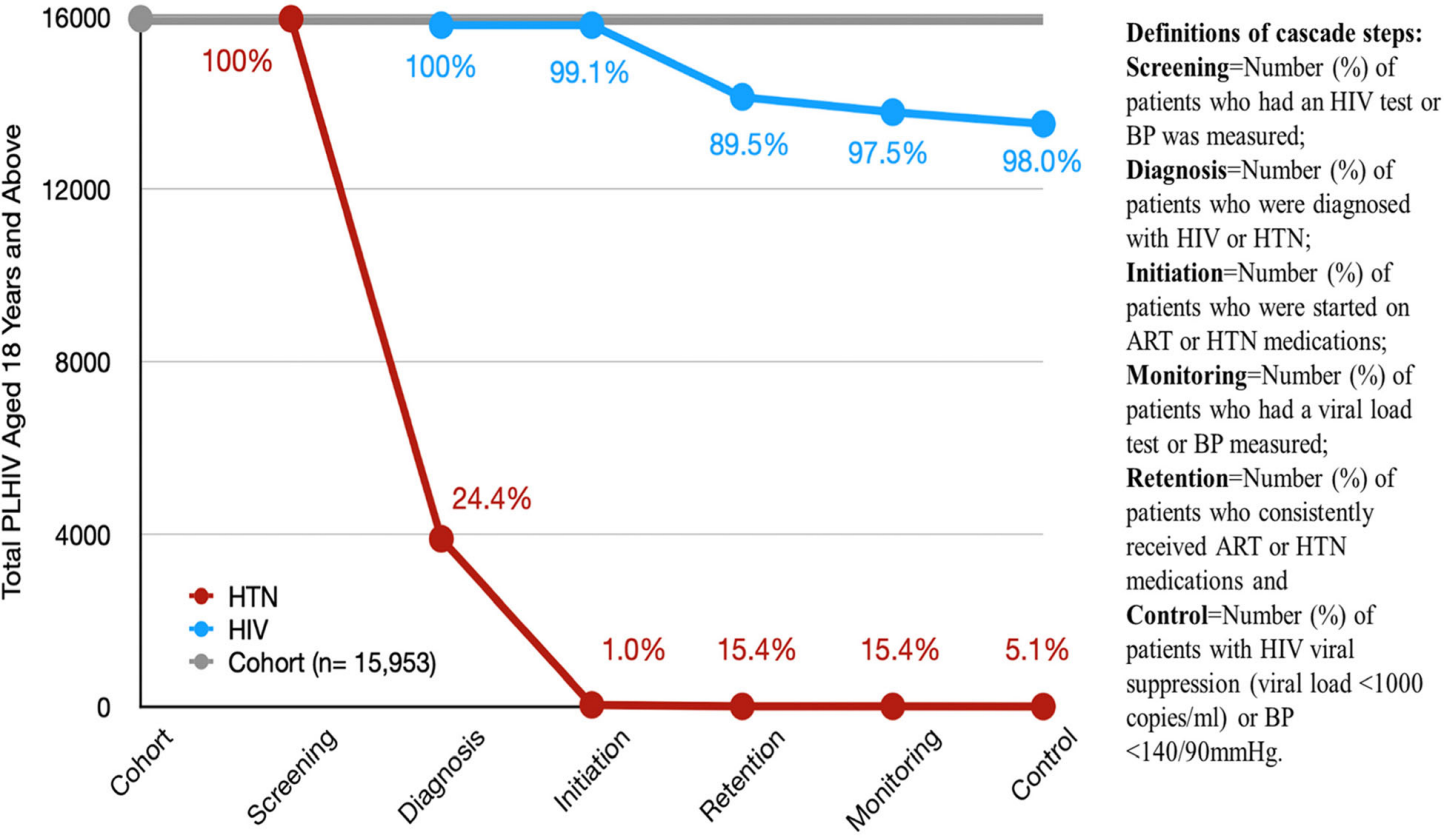
Integrated hypertension-HIV care cascades at the Mulago ISS clinic. The proportions are the percentages of participants at each particular step of the cascade as compared to the previous cascade steps

For the HIV care cascade, nearly all PLHIV 15,803 (99.1%) were initiated on ART; 14,141 (88.6%) were retained in care at the study clinic; 13,788 (97.5%) had viral load monitoring at 1 year; and 13,515 (98.0%) achieved HIV control (viral suppression) (Fig. 2).

#### Hypertension care cascade

**Fig. 3:**
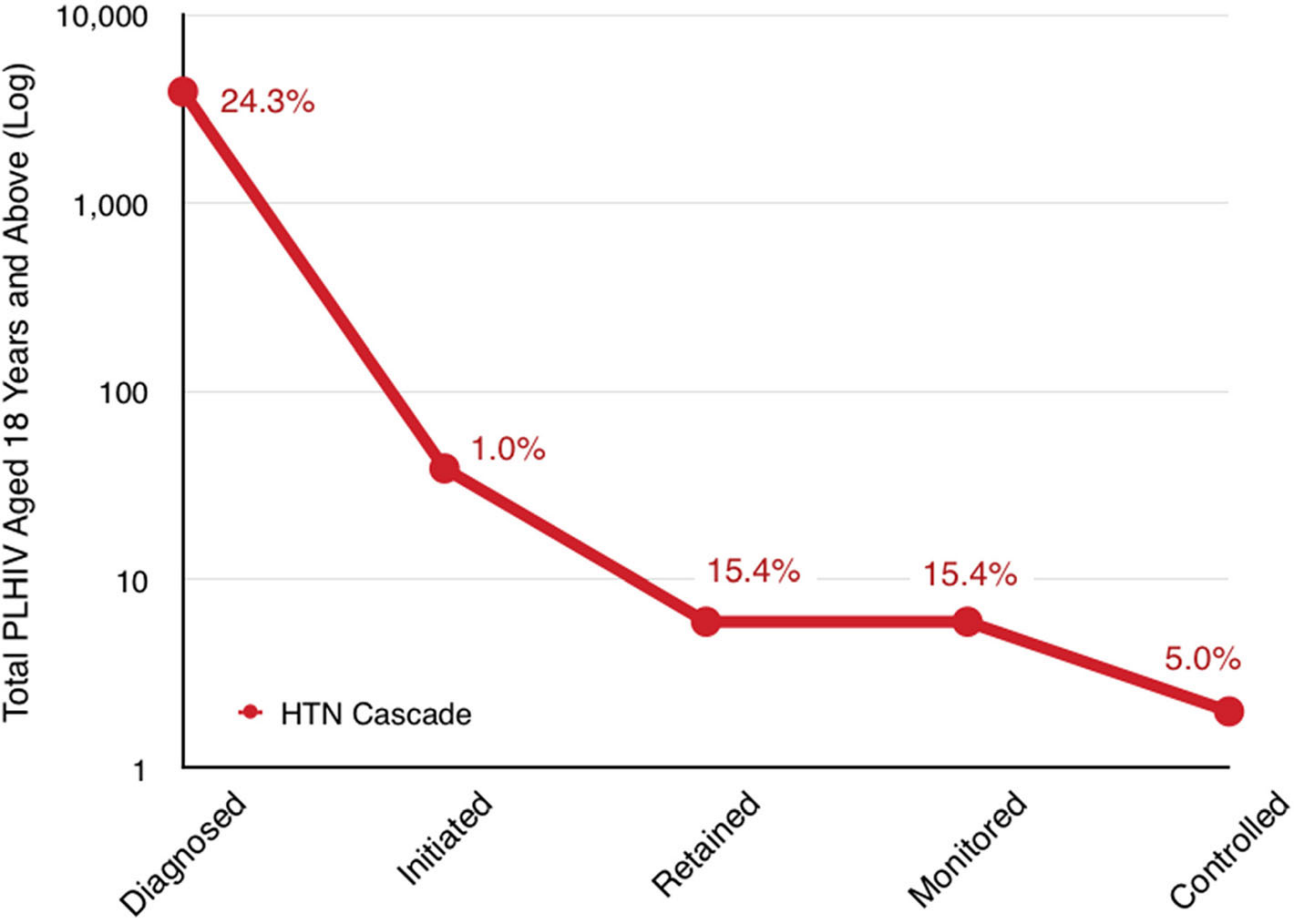
Hypertension care cascade among PLHIV who were diagnosed with HTN at the Mulago ISS clinic. The proportions are the percentages of participants at each particular step of the cascade as compared to the previous cascade steps

Of the 15,953 (100%) PLHIV who were screened for HTN, 3892 (24.4%) were diagnosed with HTN, with only 39 (1.0%) PLHIV initiated on HTN treatment. Six (15.4%) patients with both HTN and HIV were retained in care and monitored for HTN at 1 year and only two (5.0%) achieved HTN control (Figs. 2 and 3).

The qualitative findings below explain and contextualize the non-integration of HTN-HIV care as shown by the low performance of the HTN care cascade amidst a successful HIV cascade.

### Qualitative study

#### Participant characteristics

Hypertensive PLHIV (*n* = 32) and healthcare providers (*n* = 13) participated in the study. We conducted four FGDs and six IDIs with hypertensive PLHIV. Participants for the 13 KIIs included doctors, nurses, clinical officers, pharmacy technicians, HIV/HTN counselors, and HIV/HTN peer educators (Table 4). Here below we describe their perspectives regarding integrated HTN/HIV care as mapped on the COM-B model of behavior change [30, 31].

**Table 4:** Number and characteristics of participants for the qualitative study

Data collection methods	Number and category of participants	Total participants	Female ***N*** (%)	Mean age (***SD***)
Focus group discussions (FGDs) for patients	Patients who had both HTN and HIV (4 FGDs)	26	18 (69.0%)	52.0 (± 9.5)
In-depth interviews (IDIs) for patients	Patients who had both HTN and HIV (6 IDIs)	6	4 (66.7%)	44.0 (± 9.8)
Key informant interviews (KIIs) for healthcare providers	Doctor	2	1 (50.0%)	N/A
	Nurse	3	2 (66.7%)	N/A
	Clinical officer	3	2 (66.7%)	N/A
	Pharmacy technician	2	1 (50.0%)	N/A
	HIV/HTN counselor	2	1 (50.0%)	N/A
	HIV/HTN peer educator	1	1 (100%)	N/A
**Total number of participants**		**45**	**30 (66.7%)**	

#### Integration of quantitative and qualitative findings

The qualitative findings below provide the perceived barriers and facilitators of integrated HTN-HIV care from the perspectives of patients and healthcare providers. We presented the barriers and facilitators as they related to the domains of the COM-B framework. The barriers and facilitators explain why integrated HTN-HIV care either failed or succeeded according to the HIV and HTN care cascades.

Barriers to integrated HTN/HIV care related to five domains of the COM-B model including psychological capability, social opportunity, physical opportunity, automatic motivation, and reflective motivation. Facilitators emerged from five COM-B model domains of psychological capability, physical capability, physical opportunity, social opportunity, and reflective motivation (Table 5).

**Table 5:** Barriers and facilitators for integrated HTN/HIV care in the HIV clinic as related to the domains of COM-B model

COM-B domain	Barriers	Facilitators
**Psychological capability**	Patient lack of knowledge of HTN risk, complications, HTN-HIV drug interactions, and self-management	Healthcare providers have adequate knowledge of HTN screening
	Healthcare providers lack knowledge of treating HTN and HTN-HIV drug interactions	Healthcare providers and patients can leverage ART adherence support which is already being provided to PLHIV to provide adherence support for both HTN and HIV treatment
	Lack of monitoring indicators for HTN	
**Physical capability**	There were no barriers in this domain	HTN/HIV peer educators and healthcare providers have adequate skills to screen for HTN among PLHIV
		Measuring BP is easy for most of the healthcare providers including HIV peer educators
**Physical opportunity**	Lack of simple evidence-based treatment protocol for HTN care	Availability of BP machines and staff to measure and record blood pressure
	Lack of on-site HTN medications despite demand from patients and providers	
	Cost of buying anti-hypertensive medicines is high; patients cannot afford	
	Inadequate maintenance of automated BP machines at the HIV clinic	
	Lack of data collection tools and databases for HTN care	
**Social opportunity**	HTN prescriptions are mainly done by doctors; limited task shifting to clinical officers and nurses	Patients are interested in being supported by PLHIV peer educators to improve adherence to HTN and HIV treatment
**Reflective motivation**	Patients prioritize adherence to ART over HTN medications	Patients and healthcare providers have great interest in HTN/HIV integrated care
**Automatic motivation**	Lack of performance targets and review of HTN care quality	There were no facilitators in this domain

#### Perceived barriers to implementing integrated HTN-HIV care

##### Capability

*Psychological capability*

Patients’ lack of knowledge of HTN risk, complications, and self-management presented a barrier to accessing HTN treatment within the HIV clinics.

I have been with HTN and I had never screened for it and I am wondering how a tiny lady like me would have HTN. (PLHIVno 2, FGD 3).

Additionally, the asymptomatic nature of HTN limited adherence to HTN medication as patients took HTN medications only when they felt sick:

I don’t take medicines regularly and consistently, when I feel a bit sick that is when, I will buy a few. (PLHIV no 4, FGD 1).

Moreover, many PLHIV indicated they had inadequate knowledge of self-management of HTN including education on lifestyle and drug interactions:

What I would like to know is if I am to swallow both medications for HTN and HIV at the same time, will it cause any problems? (PLHIV no 8, FGD 2).

Healthcare providers lacked adequate knowledge on HTN treatment and HTN-HIV drug interactions. They identified a need to enhance their knowledge of managing HTN through training, continuing medical education, and consultation with senior practitioners. Providers believed that all cadres of healthcare providers needed training. They recommended lessons learnt in HIV care should be leveraged to improve HTN care in HIV clinics.

We need to train the nurses, peers educators, doctors, clinical officers, pharmacists and counsellors about HTN management and we should do that to the whole spectrum of cadres. We should not reinvent the wheel as we try to handle HTN. We have achieved above 97% HIV Viral suppression, we should transfer the same experience to manage HTN. (KII, Medical officer no 1).

##### Opportunity

*Physical opportunity*

Lack of HTN medications at the HIV clinic, despite demand from patients and providers, hindered the integration of HTN and HIV care. Healthcare providers prescribed HTN medications and requested patients to buy them from private pharmacies. In contrast, ART medications were always available at the clinic. All cadres of healthcare providers felt that providing free HTN medication in addition to ART would improve the HTN care cascade. They reported feeling helpless due to a lack of HTN medications to meet their clients’ needs.

The top most thing is medicine, drugs, drugs, drugs. We are doing well regarding ART, our HIV viral suppression is at 97%. For HTN, we have done nothing. We can’t buy anti-hypertensive medicines, we just prescribe and encourage our clients to go and buy. (KII, nurse no 1).

All patients across the FGDs and IDIs expressed a need to access HTN medication at no cost, as is the case for ART. Many patients revealed that they buy a few doses of HTN medicines whenever they feel unwell or have an emergency. Moreover, most patients were skeptical about the quality and efficacy of HTN medicines they would access in private pharmacies as compared to the HIV clinic where there are trained healthcare providers.

I request that someone gets us the HTN medication. When we buy it, we don’t get it in full dosage. We remember taking HTN medicine after getting an emergency. (PLHIV no 6, FGD 2).

Some private pharmacies may not give you the right medicines unlike here (HIV clinic). (IDI no 2 with a PLHIV).

Patients described lack of money and the high cost as key barriers to accessing HTN medication, and thus, some resorted to alternative treatments:

Money is the main challenge in accessing the HTN medicines. (IDI no 3 with a PLHIV).

The medicines are expensive, I have to take it on a daily basis, and I cannot buy it all at once. Sometimes I resort to using tealeaves. (PLHIV 2, FGD 3***)***

A majority of healthcare providers reported that despite having digital BP machines at the clinic, the machines were few and poorly maintained.

We use digital BP machines and the type is called Omron. I have seen a number of them are old. So, we need better machines and better cuffs (KII, nurse no 1)

Healthcare providers noted the lack of proper documentation of HTN care in the HIV medical records made patient follow-up difficult. The poor documentation led to frequent changes in HTN treatment, unlike ART where the regimes are well documented.

Clinicians record in the file when they prescribe HTN medications. For example, a clinician prescribed amlodipine for a patient and it’s not noted in the file. On the next visit, I asked the patient which drug they were taking. He said did not remember since he took it for only one month and threw away the paper. The next month, another clinician changed to Nifedipine. Onn every other visit they take a different anti-hypertensive which is really bad. (KII, medical officer no 2).

Healthcare providers reported they lacked up-to-date guidelines, HTN management protocols, standard operating procedures (SOPs), and job aides for HTN management. They reported receiving some information on WHO and MoH guidelines during CMEs; however, they also felt that they would benefit from having these guidelines, SOPs, and HTN management protocols at their workstations.

They promised to print the new guideline in the management of HTN. That’s the job aide we would be using, so we are awaiting the printing. (KII, nurse no 3).

*Social opportunity*

Nurses and clinical officers reported less involvement in HTN treatment compared to doctors. Nurses suggested they could contribute significantly to HTN treatment in the HIV clinic if they were empowered through task shifting.

There has been less involvement of nurses compared to doctors in HTN management. It is better to empower and support nurses, they are closer to patients than the doctors. (KII, Nurse no 2).

Additionally, providers noticed that leaving the treatment of HTN to only doctors increased the work load of doctors so much that they had less time to attend to more sick patients.

If you leave all HTN care to the doctor, they won’t have enough time to concentrate on complicated cases. (KII, Nurse no 3).

Task shifting the prescription of HTN medications to nurses and clinical officers would empower them to treat HTN while reducing the work load of doctors.

##### Motivation

*Reflective motivation*

Healthcare providers mentioned that patients gave less priority to HTN medicines compared to ART due to a poor understanding of the dangers of untreated HTN.

When we prescribe HTN medicines, patients do not take them seriously, they prioritize ARVs more than HTN medicines. (KII, Medical officer no 1).

Healthcare providers expressed dissatisfaction with their services since they are unable to provide HTN medicines to patients as is the case for ART. Providers mentioned they prescribe anti-hypertensive medicines for patients and encourage them to buy from private pharmacies. However, patients do not like being referred.

One patient wondered, “I have HTN and then you are referring me to another place!” They don’t want to be referred. If we had both HTN and HIV services here, we should have given them a better service. (KII, clinical officer no 1).

Providers also indicated that there were no monitoring indicators, performance targets, and systems of data collection and review for HTN unlike HIV. All PLHIV attending the clinic were screened for HTN, but screening data were not utilized since comprehensive HTN treatment was inconsistently provided.

I don’t report anywhere the clients I see who have HTN. We hope to get HTN monitoring indicators and performance targets as the project kicks off, but now we have those for HIV care but not for HTN. (KII, medical officer no 2).

#### Perceived facilitators of implementing integrated HTN-HIV care

##### Capability

*Psychological capability*

Healthcare providers mentioned that ART adherence counseling is always emphasized: Building on adherence counseling for ART is an opportunity to integrate the management of HTN into HIV care. Healthcare providers noted that adherence counseling for both ART and HTN medications would enhance the psychological capability of patients to adhere to both treatments.

We shall not emphasize only adherence to ART: we shall also include adherence counselling for HTN medications. (KII, nurse no 3).

*Physical capability*

A majority of healthcare providers reported having the necessary skills to measure blood pressure. This facilitated screening for HTN among PLHIV at the HIV clinics.

Screening for HTN is a simple procedure. We need a comfortable chair, table and a BP machine, which should be well maintained. Patients should be calm (KII, nurse no 2).

##### Opportunity

*Social opportunity*

Healthcare providers mentioned some social opportunities for integrating the management of HTN into HIV care including task shifting, empowering HIV peer counselors to screen and nurses to treat HTN. This form of task shifting is already being implemented for HIV care in the clinics. Healthcare providers suggested HIV peer educators should receive targeted capacity-building for HTN care. This would facilitate effective task shifting since patients consult and trust the HIV peer educators.

Our peer educators screen for BP and they do it well. Majority of our patients trust their peers more than us; if the peer educators don’t have the right information, they will give misleading information. (KII, Nurse no 1).

*Reflective motivation*

Patients’ experiences about HTN screening were generally positive as they reported having their BP measured at every clinic visit. Building on this opportunity would foster integrating HTN management into HIV care.

I express my gratitude to the health workers because when we come to the HIV clinic they take our BP measurements and they give us advice when the BP is high. (PLHIV no 6, FGD 3).

All patients supported the idea of receiving HTN and HIV care together at the same clinic visit. In addition, patients reported that improving access to HTN medicine would improve adherence.

If HTN treatment was available here, I wouldn’t want to go to any other place because it would make life easier. We would be receiving both our HIV and HTN medicines from the same place. In that way, I would use the same transport to come here and receive both treatments. (PLHIV no 5, FGD 1).

*Automatic motivation*

There were mixed reactions about patients being supported by peer educators in managing HTN. Most patients welcomed peer support for HTN and HIV treatment, reporting that hearing from a client who has controlled both conditions would give them encouragement:

It would be good to have someone who reminds me all the time as well as counsels me. I would get courage from that person to adhere to treatment especially if her/his HTN and HIV were initially uncontrolled but is now well off. (PLHIV no 6, FGD 4).

Patients who had spent longer time on ART reported that they had the automatic motivation to manage HIV and HTN and may not require peer support. These patients expressed knowledge of their condition, understood the importance of treatment adherence, and were motivated to adhere to both HTN and HIV treatments.

For us, who have spent some time on these medicines (ARVs), we already know what we are supposed to do. We don’t need someone reminding us, we can do this ourselves because we know what time we are supposed to swallow these medicines and the importance of adhering. (PLHIV no 3, FGD 1).

## Discussion

In this explanatory sequential mixed methods study, we compared the HIV and HTN care cascades among PLHIV. The goal was to identify gaps in HTN care within a successful HIV clinic which could be targeted in future interventions. We then used qualitative inquiry guided by the COM-B model to explain findings of the HIV and HTN cascades. To this effect, we explored the perceived barriers and facilitators of integrating HTN care into an urban HIV program in East Africa from the perceptive of patients and providers [27].

This urban HIV clinic is highly successful in terms of HIV treatment cascade metrics. We established that the HIV care cascade achieved the UNAIDS target of initiating more than 95% of all PLHIV on ART. In addition, approximately 90% of all PLHIV initiated on ART were retained at 1 year while 98% of all PLHIV diagnosed achieved viral suppression [23]. Despite the exceptional success in HIV care, the HTN care cascade among PLHIV in the same clinic was dismal. Despite routine BP screening for all PLHIV ≥18 years old at each visit and diagnosing nearly a quarter (24.4%) with HTN, only 1% of all hypertensive PLHIV were started on treatment for HTN. Retention in HTN care and BP control were consequently suboptimal at 15.4% and 5%, respectively. Poor integration of HTN and HIV care has also been observed in rural clinics of Eastern Uganda and other parts of SSA [26, 29]. This disparity in care for HTN even in a setting of excellent HIV services is problematic in a country like Uganda, where HTN is by far the leading risk factor for cardiovascular disease. In Uganda, for example, high blood pressure contributes four times higher estimated CVD disability-adjusted life years (DALYs) compared to high LDL cholesterol [35].

Among PLHIV aged 18 years and above, HTN was associated with older age, being male, longer duration of ART, and ART containing nevirapine. These findings are consistent with other studies in SSA [2, 3, 36, 37]. The association with ART duration and older ART medicines may be partly confounded by age [38, 39]. However, multivariate analysis ruled out this confounding and emphasized a need for HTN care among aging PLHIV.

Our findings complement growing literature that supports the integration of care for NCDs like HTN within existing HIV services by leveraging the huge gains attained by HIV programs [13–17]. The HIV program has empowered healthcare providers to implement guidelines and care algorithms. Additionally, the HIV program has well-developed systems for linking patients to care, treatment adherence counseling and support, medication access, and monitoring and evaluation. These strategies have succeeded in keeping individuals in care with high adherence to medication and should be leveraged to integrate the management of HTN into HIV care.

Most hypertensive PLHIV expressed their desire for HTN/HIV peer educators to provide psychosocial support aimed at improving treatment adherence and retention for both conditions [40–43]. An integrated HTN/HIV program should leverage the existing HIV peer network to improve HTN care among PLHIV [44].

Moreover, healthcare providers and patients have an opportunity to leverage existing ART adherence support, which is routinely offered to all PLHIV. Healthcare providers and patients reaffirmed the benefits of high-quality ART adherence support services at the clinic and added that HTN medication adherence could easily be integrated in the same manner. To address patient-related barriers to HTN treatment, adherence counseling and support for both ART and HTN should be integrated to educate patients on risks and complications of HTN, possible drug interaction between ART and HTN medications and self-management. PLHIV were hopeful to achieve greater adherence to both HTN medications and ART if delivered as an integrated service [45].

Moreover, both PLHIV and healthcare providers expressed interest in integrated HTN/HIV care. Evidence from other SSA settings shows that integrated HTN/HIV rather than vertical programs are more cost effective and more likely to achieve HTN control [17, 21, 46, 47].

All healthcare providers and PLHIV peer educators felt that measuring BP was an easy procedure. In addition, the cascade analysis showed that all PLHIV had their BP measured at least once a year. This clinic was already screening all PLHIV for HTN during each clinic visit even before our study started. BP measurement provides a great opportunity to improve the HTN care cascade since it is the entry point to HTN care [17, 48]. Shifting the role of BP measurement to HIV peer counselors could lead to even greater efficiency and provide an opportunity for counseling; however, this will require training and simple, standardized protocols.

Generally, PLHIV expressed inadequate knowledge of HTN risk, complications, and self-management, and they prioritized adherence to ART over HTN medicines. Studies in Rwanda and South Africa showed similar findings that hypertensive PLHIV lacked knowledge on risk factors and complications of HTN [17, 48, 49]. Empowering patients with knowledge may provide an opportunity for better treatment adherence and may facilitate self-care [40].

Some healthcare providers expressed inadequate knowledge of HTN treatment algorithms and drug interactions. Training of healthcare providers on HTN management is required for a robust integrated HTN/HIV program [28, 50]. Moreover, healthcare providers lacked simple treatment protocols for HTN within an HIV clinic. HTN/HIV integrated programs need to leverage the WHO HEARTS technical packages for HTN control which provide simple and stepwise evidence-based treatment protocols for HTN in the primary healthcare setting [51–55].

Poor access to HTN medications and BP machines have been cited as key barriers to HTN control in SSA [27]. In our study, it was evident from patients and healthcare providers that this inevitably contributed to the poor HTN cascade at Mulago ISS. Currently, patients are asked to pay for HTN medicines out of pocket, but cannot afford them [56]. Simply providing access to effective medicines for HTN at little or no cost to the patient may greatly improve HTN cascade metrics and complement the expanded access to ART. This will require countries to adopt lessons of the HIV program to HTN management by developing effective HTN treatment protocols and consistently procuring effective HTN medications.

### Limitations of the study

We interviewed patients who had been on ART for at least 1 year and healthcare providers who had provided HIV care for at least 2 years. These participants might have felt strongly about the importance of HTN-HIV integration. This study was conducted at a single, unique HIV clinic; however, Mulago ISS is the largest HIV clinic in Uganda providing ART to over 16,500 PLHIV. HIV services at the clinic are provided in line with the consolidated guidelines for the prevention and treatment of HIV and AIDS in Uganda [19]. In addition, the clinic serves clients from all parts of the country since it is located at and linked to the National Referral Hospital. Given the breadth of the study population, we believe that the findings of this study may be generalizable to HIV-endemic LMIC settings.

## Conclusion

The prevalence of HTN among PLHIV at a large, urban HIV clinic is high but the cascade of care for HTN is poor. There is a need to engage key stakeholders to inform the designing of contextually appropriate and targeted interventions for HTN/HIV integration, to close these gaps in HIV-endemic LMICs. Future research should address clinical outcomes, cost, and cost effectiveness of integrated HTN/HIV services [15, 57].

## Data Availability

The datasets used and/or analyzed during the current study are available from the corresponding author on reasonable request.

## Abbreviations

COM-B: Capability, Opportunity, Motivation and Behavior
HTN: Hypertension
PLHIV: Persons living with HIV
CVD: Cardiovascular disease
NCD: Non-communicable disease
ART: Antiretroviral therapy
WHO: World Health Organization
MoH: Ministry of Health
FGD: Focus group discussion
KII: Key informant interview
IDI: In-depth interview
LMICs: Low- and middle-income countries
